# A single quantum dot-based nanosensor with multilayer of multiple acceptors for ultrasensitive detection of human alkyladenine DNA glycosylase†
†Electronic supplementary information (ESI) available. See DOI: 10.1039/c9sc02137j


**DOI:** 10.1039/c9sc02137j

**Published:** 2019-08-06

**Authors:** Chen-chen Li, Wan-xin Liu, Juan Hu, Chun-yang Zhang

**Affiliations:** a College of Chemistry, Chemical Engineering and Materials Science , Collaborative Innovation Center of Functionalized Probes for Chemical Imaging in Universities of Shandong , Key Laboratory of Molecular and Nano Probes , Ministry of Education , Shandong Provincial Key Laboratory of Clean Production of Fine Chemicals , Shandong Normal University , Jinan 250014 , China . Email: cyzhang@sdnu.edu.cn ; Email: juanhu@sdnu.edu.cn ; Fax: +86 0531-82615258 ; Tel: +86 0531-86186033

## Abstract

Base excision repair (BER) is an important DNA repair pathway involved in the maintenance of genome stability. As the initiator of BER, DNA glycosylase can remove a damaged base from DNA through cleaving the N-glycosidic bond between the sugar moiety and the damaged base. Accurate quantification of DNA glycosylase is essential for the early diagnosis of various human diseases. However, conventional methods for DNA glycosylase assay usually suffer from poor sensitivity and complex probe design. Herein, we develop a single quantum dot-based nanosensor with multilayer of multiple acceptors for ultrasensitive detection of human alkyladenine DNA glycosylase (hAAG) using apurinic/apyrimidinic endonuclease 1 (APE1)-assisted cyclic cleavage-mediated signal amplification in combination with the DNA polymerase-assisted multiple cyanine 5 (Cy5)-mediated fluorescence resonance energy transfer (FRET). The presence of hAAG induces the cleavage of the hairpin substrate, generating a trigger. The resultant trigger can hybridize with a probe modified with an AP site, initiating the APE1-mediated cyclic cleavage to produce a large number of primers. The primers can subsequently initiate the polymerase-mediated signal amplification with a biotin-modified capture probe as the template, generating the biotin-/multiple Cy5-labeled double-stranded DNAs (dsDNAs). The resultant dsDNAs can assemble onto the QD surface to form the QD-dsDNA-Cy5 nanostructure, leading to efficient FRET from the QD to Cy5 under the excitation of 405 nm. In contrast to the typical QD-based FRET approaches, the assembly of multilayer of multiple Cy5 molecules onto a single QD significantly amplifies the FRET signal. We further verify the FRET model with one donor and multilayered acceptors theoretically and experimentally. This single QD-based nanosensor can sensitively detect hAAG with a detection limit of as low as 4.42 × 10^–12^ U μL^–1^. Moreover, it can detect hAAG even in a single cancer cell, and distinguish the cancer cells from the normal cells. Importantly, this single QD-based nanosensor can be used for the kinetic study and inhibition assay, and it may become a universal platform for the detection of other DNA repair enzymes by designing appropriate DNA substrates.

## Abstract 2

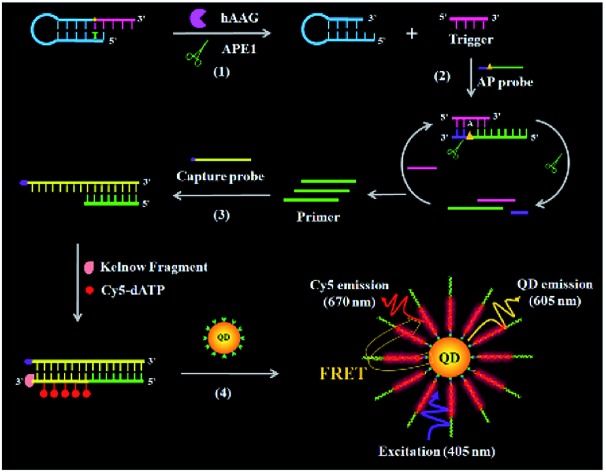
We develop a single quantum dot-based nanosensor with multilayer of multiple acceptors for ultrasensitive detection of human alkyladenine DNA glycosylase.

## Introduction

The genetic information of eukaryotes is stored in DNA whose maintenance and stability are vital to the organism. However, the genomic DNA is constantly exposed to various endogenous and environmental threats (*e.g.*, reactive radical species, toxins, and radiation), causing a diversity of damaged bases, lesions, mismatches and base-pair modifications in the genome,^1^ eventually leading to genomic instability and cancers.^2^,^3^ The base excision repair (BER) pathway acts throughout the cell cycle to remove the damaged bases from DNA. BER is initiated by DNA glycosylases that recognize and catalyse the cleavage of the damaged/mismatched bases, generating an apurinic/apyrimidinic (AP) site. The repair process is completed by the action of AP endonucleases, deoxyribophosphodiesterases, DNA polymerases and DNA ligases.^4^–^6^ BER enzymes (*e.g.*, DNA glycosylases) play an important role in the repair of DNA lesions and have been associated with both individual and population disease susceptibility.^7^ In addition, abnormal DNA glycosylases are associated with a variety of diseases such as cancer,^8^,^9^ cardiovascular disease,^10^ neurological disease and inflammation,^11^ suggesting the important role of DNA glycosylases in cancer diagnosis and treatment.

So far, a series of methods have been developed for the detection of DNA glycosylase.^12^–^20^ Gel-electrophoresis coupled with radioactive labelling is the most general method, but it suffers from time-consuming procedures, poor sensitivity, and hazardous radiation.^12^ High-performance liquid chromatography needs tedious DNA fragmentation and expensive instrumentation.^13^ Magnetic nanoparticle-based separation techniques involve a long analysis time and complicated procedures.^14^ Gold nanoparticle-based colorimetric assays enable visual detection of DNA glycosylase, but they require complicated procedures for the preparation and modification of gold nanoparticles.^15^ Luminescence assays need additional chemical reagents which increases the complexity of the experiments.^16^ Fluorescence methods take advantage of either DNA probes labelled with a fluorophore and a quencher^17^,^18^ or artificial fluorescent nucleotide analogs (*e.g.*, pyrene, 2-aminopurine, and pyrrolo-dC)^19^,^20^ to detect thymine DNA glycosylase (TDG),^18^ uracil DNA glycosylase (UDG),^19^ and human 8-oxoguanine DNA glycosylase (hOGG1),^20^ but few approaches are available for human alkyladenine DNA glycosylase (hAAG) assay.^17^ Unlike other DNA glycosylases that are specific for a particular type of damaged base, hAAG excises a diversity of substrate bases damaged by alkylation and deamination (*e.g.*, 3-methyladenine, 7-methylguanine, 1,*N*^6^-ethenoadenine, and hypoxanthine).^21^ The hAAG cleaves the N-glycosidic bond between the sugar and the damaged base (see ESI, Fig. S1†), and the resulting abasic nucleotide is excised and replaced with a normal nucleotide by the sequential action of endonuclease, polymerase and DNA ligase.^21^ Previous research demonstrates that hAAG activity in peripheral blood mononuclear cells from lung cancer patients is higher than in normal people.^22^ Moreover, the high expression of hAAG may induce frameshift mutagenesis and microsatellite instability by binding to and stabilizing one and two base-pair loops and shielding them from repair in the presence and absence of the DNA mismatch repair pathway, eventually leading to a high risk of cancer.^23^ Therefore, the accurate detection of hAAG activity is essential for biomedical research and clinical diagnosis.

Semiconductor quantum dots (QDs) exhibit unique optical and physical properties (*e.g.*, high brightness, high quantum yield, good stability against photobleaching, narrow emission bands and size-tunable emission spectra) that are not shared by organic dyes and fluorescent proteins, and their dimensions are comparable to those of biomolecules.^24^–^26^ QDs have found wide applications in imaging, sensing, drug delivery and biomedical research.^27^–^32^ Recently, the combination of QDs with single-molecule/particle detection^33^–^37^ enables the detection of nucleic acids, proteins, and even small molecules with extremely high sensitivity, low sample consumption, rapidity, and simplicity.^38^–^40^ In this research, we develop a single QD-based nanosensor with multilayer of multiple acceptors for ultrasensitive detection of hAAG using apurinic/apyrimidinic endonuclease 1 (APE1)-assisted cyclic cleavage-mediated signal amplification in combination with the DNA polymerase-assisted multiple Cy5-mediated FRET. We designed a hairpin probe with a hypoxanthine base (I) modified in its stem as the substrate of hAAG. The presence of hAAG induces the cleavage of the hairpin substrate, generating a trigger which can hybridize with a probe modified with an AP site to initiate the cyclic cleavage for the generation of abundant primers. The resultant primers can subsequently initiate the polymerase-mediated signal amplification to produce the biotin-/multiple Cy5-labeled double-stranded DNAs (dsDNAs) which can assemble onto the QD surface to form the QD-dsDNA-Cy5 nanostructure, leading to efficient FRET from the QD to Cy5 under the excitation of 405 nm. This single QD-based nanosensor can sensitively detect hAAG with a detection limit of as low as 4.42 × 10^–12^ U μL^–1^. Moreover, it can detect hAAG even in a single cancer cell, and distinguish the cancer cells from the normal cells.

## Results and discussion

### Principle of the hAAG assay

**Scheme 1:**
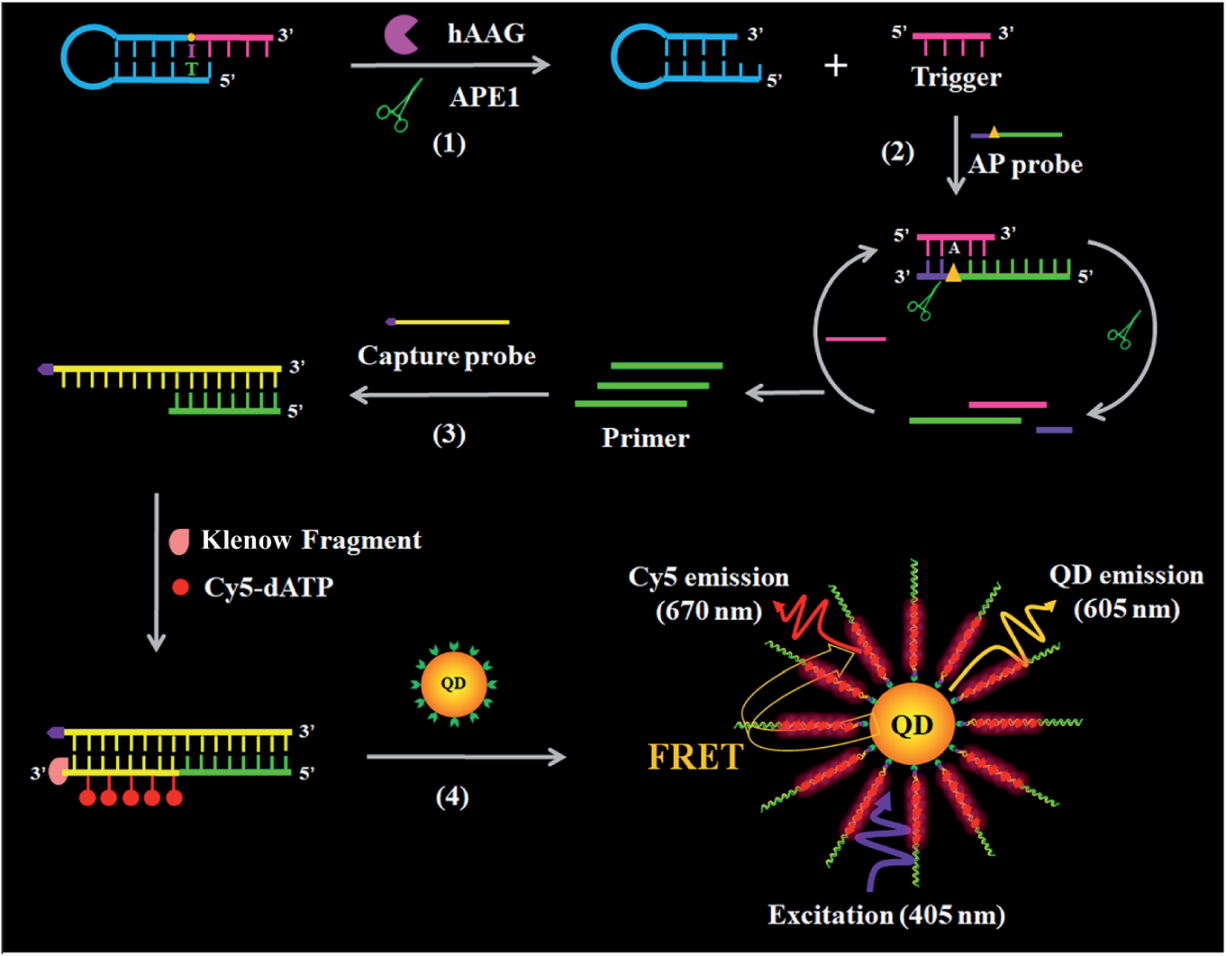
Principle of the single QD-based nanosensor with multilayer of multiple Cy5 molecules for the hAAG activity assay. This assay involves four steps: (1) hAAG-actuated hypoxanthine-excision repair reaction, (2) APE1-induced strand displacement amplification (SDA), (3) DNA polymerase-assisted amplification accompanied by the incorporation of multiple Cy5 molecules, and (4) the assembly of biotin-/multiple Cy5-labeled dsDNAs onto the surface of the QD, resulting in efficient FRET from the QD to Cy5.

The principle of the single QD-based nanosensor with multilayer of multiple acceptors for hAAG activity is illustrated in Scheme 1. The reaction system consists of a hairpin probe, an AP probe, and a capture probe. We designed a hairpin probe as the substrate of hAAG. The stem of the hairpin probe contains two complementary strands, with a hypoxanthine base (I) modified in the longer stem (blue + pink color, Scheme 1), which is mismatched with thymine (T) in the complementary strand (blue color, Scheme 1). We designed an AP probe with an AP site (green + purple color, Scheme 1) at the 23rd base from the 5′ end. The AP probe is paired with the trigger (pink color, Scheme 1) generated by the cleavage of the hairpin probe for the initiation of isothermal strand displacement amplification (SDA)^41^,^42^ in the presence of APE1. In addition, we designed a capture probe (yellow color, Scheme 1) which can hybridize with the primer (green color, Scheme 1) for the initiation of DNA polymerase-assisted amplification. To prevent the nonspecific amplification, we modified the 3′ termini of all probes with NH_2_.

This assay involves four steps: (1) hAAG-actuated hypoxanthine excision repair reaction, (2) APE1-mediated SDA, (3) DNA polymerase-assisted amplification and the incorporation of multiple Cy5 molecules, and (4) the assembly of biotin-/multiple Cy5-labeled dsDNAs onto the QD surface, resulting in efficient FRET from the QD to Cy5. In the presence of the hAAG, APE1 enzyme and hairpin probe, hAAG recognizes the I/T base pairs and cleaves the N-glycosidic bond between the sugar and the hypoxanthine base, releasing the hypoxanthine base to form an AP site.^43^,^44^ Then APE1 cleaves the AP site, leading to the break of the hairpin probe into two portions (*i.e.*, a trigger and a stable stem-loop DNA fragment) (see ESI, Fig. S1C†). The resultant triggers (pink color, Scheme 1) can hybridize with the AP probes to form the AP probe/trigger dsDNAs. Subsequently, the APE1 enzyme induces cyclic cleavage of dsDNAs, releasing the triggers and a large number of primers with 3′-OH (green color, Scheme 1). The released primers can initiate polymerization with the biotinylated capture probe as the template in the presence of the Klenow fragment, Cy5-dATP, dCTP, dGTP and dTTP, generating stable dsDNAs with the incorporation of multiple Cy5 molecules. These biotin-/multiple Cy5-labeled dsDNAs can self-assemble onto the QD surface *via* specific biotin–streptavidin binding to form the QD-dsDNA-Cy5 nanostructure. Under the excitation of 405 nm, efficient FRET occurs with the 605QD as the donor and Cy5 as the acceptor (see ESI, Fig. S2†), and the Cy5 signals can be simply measured using a total internal reflection fluorescence (TIRF) microscope for the quantification of hAAG activity, while in the absence of hAAG, the hypoxanthine base cannot be cleaved, and no trigger is generated. Consequently, neither APE1-mediated SDA nor DNA polymerase-assisted amplification reaction occurs, and no Cy5 signal is observed. Notably, this assay has four significant characteristics: (1) APE1 can specifically cleave the AP site in the DNA duplex of both the hairpin probe and AP probe/trigger dsDNA, initiating the APE1-mediated SDA for the release of a large number of primers; (2) the capture probe can specifically hybridize with the primer, forming dsDNA which may function as the template for the amplification, leading to the incorporation of multiple Cy5 molecules into the resultant dsDNA and eventually the formation of the QD-dsDNA-Cy5 nanostructure; (3) in the single QD-based FRET nanosensor, the QD functions not only as a FRET donor but also as a local nanoconcentrator to assemble multiple Cy5 acceptors. In contrast to the typical QD-based FRET approaches with a single donor–acceptor pair,^38^ the assembly of biotin-/multiple Cy5-labeled dsDNAs onto a single QD leads to the formation of the multilayer of multiple Cy5 molecules in the QD-dsDNA-Cy5 nanostructure, significantly improving the FRET signals; (4) the near-zero background signal results from the specific recognition and cleavage of the hairpin probe substrate by hAAG and a high signal-to-noise ratio of single-molecule detection. Therefore, this single QD-based nanosensor with multilayer of multiple acceptors can be applied for sensitive detection of DNA glycosylase.

### Validation of the assay

**Fig. 1:**
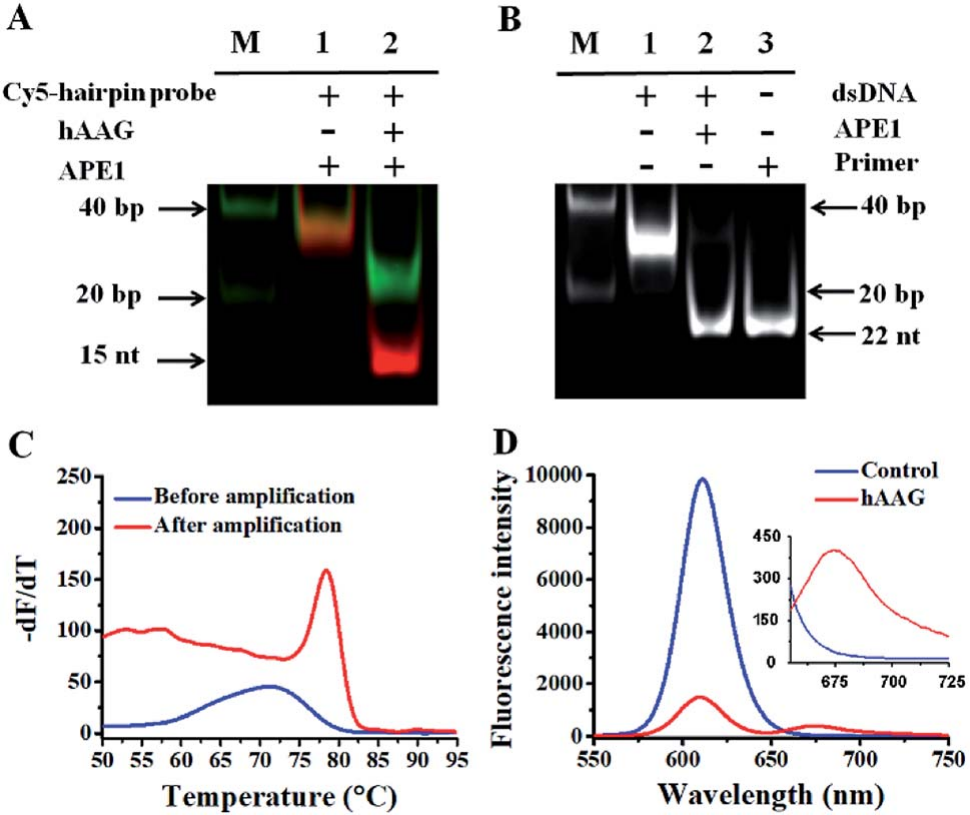
(A) PAGE analysis of the hAAG-mediated cleavage of Cy5-hairpin probes with SYBR Gold as the indicator. Lane 1, in the absence of hAAG; lane 2, in the presence of hAAG. (B) PAGE analysis of the APE1-mediated SDA with SYBR Gold as the indicator. Lane 1, in the absence of APE1; lane 2, in the presence of APE1; lane 3, with the synthesized primer as a reference. (C) Variance of the –d*F*/d*T* with the temperature before (blue line) and after (red line) DNA polymerase-assisted amplification. *F* is the fluorescence intensity and *T* is the temperature. (D) Measurement of 605QD and Cy5 fluorescence emission spectra in the absence (control, blue line) and presence (red line) of hAAG. The inset shows magnified fluorescence spectra from 655 to 725 nm. The hAAG concentration is 0.1 U μL^–1^.

In order to verify the feasibility of this assay, we performed gel electrophoresis, DNA melting temperature experiments, and fluorescence measurements, respectively (Fig. 1). We used a Cy5-labeled hairpin probe to perform the hAAG-actuated hypoxanthine excision repair reaction (Fig. 1A). In the absence of hAAG, only a 54-nt band of the Cy5-hairpin probe is observed with the co-localization of SYBR Gold and Cy5 (Fig. 1A, lane 1), indicating no occurrence of cleavage reaction. In the presence of hAAG, the Cy5-hairpin probe is cleaved, generating a 15-nt band of the Cy5-labeled trigger (Fig. 1A, lane 2, red color) and a 38-nt band of the stem-loop DNA fragment (Fig. 1A, lane 2, green color), indicating that hAAG can recognize the I/T base pair and specifically excise the hypoxanthine with the assistance of APE1.

**Fig. 2:**
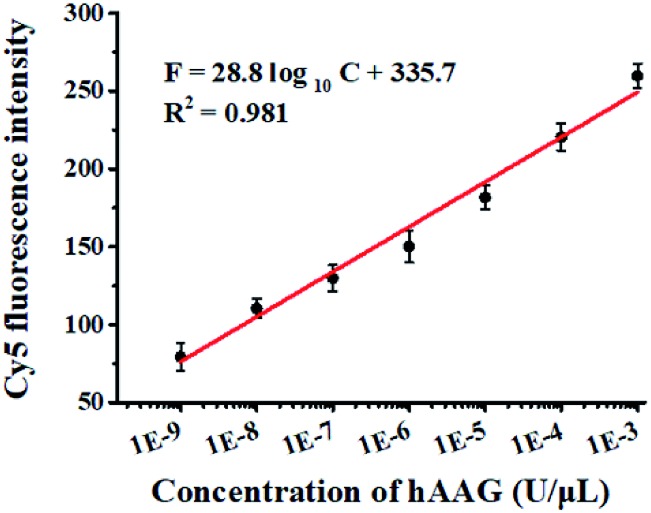
Linear relationship between Cy5 fluorescence intensity and the logarithm of hAAG concentration in the range from 1 × 10^–9^ to 1 × 10^–3^ U μL^–1^. Error bars represent standard deviations of three experiments.

The cleavage of the AP site in the dsDNA by APE1 is verified by gel electrophoresis (Fig. 1B). The hybridization of the trigger with the AP probe leads to the formation of the trigger/AP probe dsDNA (Fig. 1B, lane 1). The APE1 can cleave the AP site of dsDNA, producing a 22-nt primer (Fig. 1B, lane 2) with the same length as the synthesized primer (Fig. 1B, lane 3), indicating the occurrence of APE1-medicated SDA. To confirm whether the hybridization of the primers with the capture probes can initiate the Klenow fragment polymerase-assisted amplification, we measured the melting curves of products (Fig. 1C). The melting temperature of products is 78 °C after amplification (Fig. 1C, red line), much higher than that before amplification (71 °C; Fig. 1C, blue line), suggesting the occurrence of the Klenow fragment polymerase-assisted amplification reaction. We further used fluorescence spectroscopy to verify the feasibility of this assay (Fig. 1D). No Cy5 signal is detected in the control without hAAG (Fig. 1D, blue line), indicating no FRET from the QD to Cy5 in the absence of hAAG. In contrast, in the presence of hAAG, a distinct Cy5 signal is observed, accompanied by the decrease of the QD signal (Fig. 1D, red line), suggesting efficient FRET from the QD to Cy5 as a result of the formation of the QD-dsDNA-Cy5 nanostructure. Moreover, the Cy5 fluorescence intensity enhances with increasing concentration of hAAG (Fig. 2). In the logarithmic scale, the Cy5 fluorescence intensity exhibits a linear correlation with the concentration of hAAG in the range from 1 × 10^–9^ to 1 × 10^–3^ U μL^–1^. The regression equation is *F* = 28.8 log_10_ *C* + 335.7 (*R*^2^ = 0.981), where *C* represents the concentration of hAAG (U μL^–1^) and *F* represents the Cy5 fluorescence intensity. The detection limit is calculated to be 8.98 × 10^–10^ U μL^–1^ based on the principle of the control group plus three times the standard deviation. Notably, the Cy5 molecules can covalently bind to the QD surface only through the DNA polymerase-assisted amplification reaction and specific biotin–streptavidin binding in the presence of hAAG, without any nonspecific absorption of Cy5-dATP on the surface of streptavidin-coated QDs due to the presence of biotin-labeled probes (*e.g.*, the capture probe in Scheme 1 and the biotinylated random sequence in Fig. S3, see ESI†).

### Calculation of FRET efficiency of the single QD-based nanosensor

In the single QD-based nanosensor, FRET leads to the simultaneous quenching of the QD donor emission and sensitization of the Cy5 acceptor emission. The FRET efficiency (*E*) can be quantified based on eqn (1)^25^1
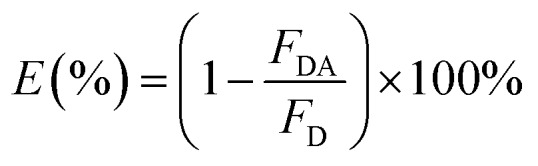
where *F*_DA_ is the 605QD fluorescence intensity in the presence of the Cy5 acceptor, and *F*_D_ is the 605QD fluorescence intensity in the absence of the Cy5 acceptor. Under the optimal experimental conditions (see ESI, Fig. S4–S7†), the FRET efficiency is calculated to be 84.9% according to eqn (1). This value is close to that (85.3%) obtained by the single QD-based nanosensor (see ESI, Fig. S4†). Such a high FRET efficiency is reasonable theoretically. In the single QD-based nanosensor, the FRET efficiency (*E*) can be calculated based on eqn (2)^25^2
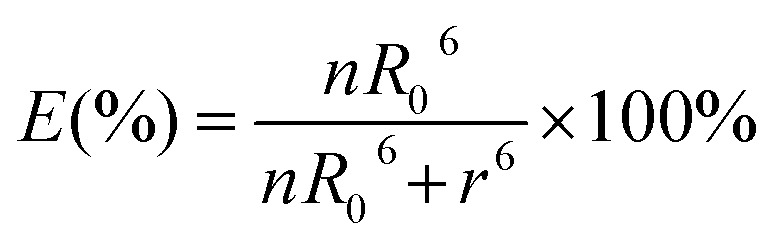
where *R*_0_ is the Förster distance, *n* is the average number of acceptor molecules interacting with one donor, and *r* is the distance from the acceptor to the donor centre. The Förster distance (*R*_0_) is estimated to be 7.7 nm for the 605QD/Cy5 pair.^40^ We calculated the separation distance between the 605QD donor and the Cy5 acceptor in the FRET-based nanosensor. In theory, five Cy5-dATP can be added to the end of each primer as a result of the DNA polymerase-assisted amplification. The distances between Cy5 molecules in the extended primer and the surface of streptavidin-coated QD are estimated to be 1.36 nm, 3.06 nm, 4.76 nm, 6.46 nm, and 7.82 nm, respectively, based on the assumption that the average length of the nucleotide in dsDNA is 0.34 nm. Taking into account the radius of the streptavidin-functionalized QD (7.5–10 nm) (see ESI, Fig. S8†), the corresponding QD-Cy5 separation distances are calculated to be 11.36 nm (∼1.5 × *R*_0_) for Cy5 in position-1, 13.06 nm (∼1.7 × *R*_0_) for Cy5 in position-2, 14.76 nm (∼1.9 × *R*_0_) for Cy5 in position-3, 16.46 nm for Cy5 in position-4, and 17.82 nm for Cy5 in position-5. To obtain efficient FRET, the structure can be regarded as a QD-Cy5-Cy5-Cy5 configuration with Cy5 molecules located at positions 1–3. The value of *n* = 36 is further confirmed by both the FRET experiments (see ESI, Fig. S4†) and the measurement of the number of biotin-/multiple Cy5-labeled dsDNAs per 605QD using fluorescence emission spectra (see ESI, Fig. S9†). When *R*_0_ is 7.7 nm and *n* is 36, the FRET efficiency (*E*) for a single QD with multiple acceptors can be calculated to be 77.7% for Cy5 in position-1 (*i.e.*, *E*_1_ = 77.7%), 60.2% for Cy5 in position-2 (*i.e.*, *E*_2_ = 60.2%), and 42.0% for Cy5 in position-3 (*i.e.*, *E*_3_ = 42.0%) based on eqn (2). In the QD-Cy5-Cy5-Cy5 configuration, the theoretical total FRET efficiency (*E*_th_) can be obtained from the individual single-pair FRET efficiencies (*i.e.*, *E*_1_, *E*_2_, and *E*_3_) based on eqn (3)^45^3
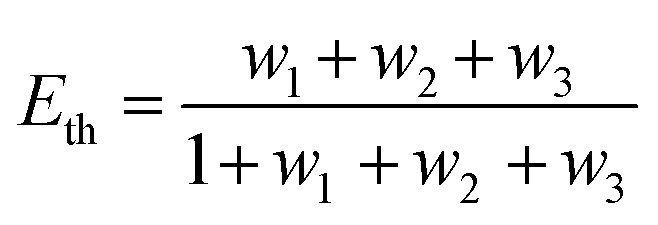
where *w*_*i*_ = *E*_*i*_/(1 – *E*_*i*_). In this QD-dsDNA-Cy5 nanosensor with multilayered Cy5 acceptors, the total efficiency (*E*_th_) is calculated to be 85.1%, consistent with the value obtained by fluorescence spectroscopy measurement (84.9%) and the value obtained by the single QD-based nanosensor (85.3%).

We further investigated the distribution of energy in this QD-Cy5-Cy5-Cy5 configuration. The total FRET efficiency can be derived from three QD-Cy5 subsystems (*i.e.*, the QD/Cy5 pairs with Cy5 in position-1, Cy5 in position-2, and Cy5 in position-3, respectively) based on eqn (4)–(6),^46^4
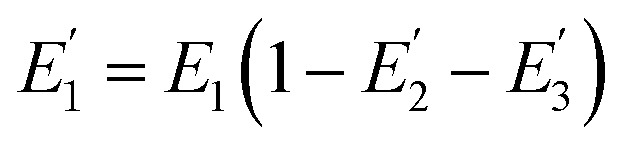

5
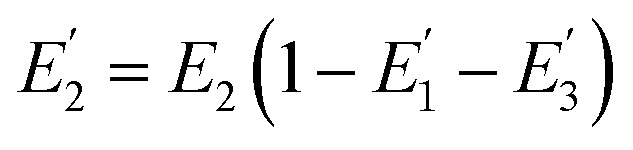

6
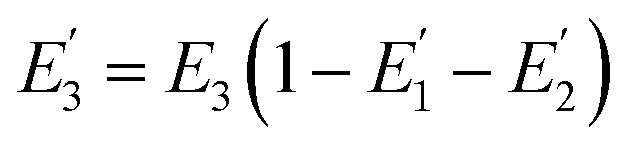
where 
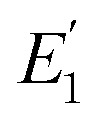
 is the FRET efficiency of the QD/Cy5 pair with Cy5 in position-1, 
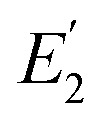
 is the FRET efficiency of the QD/Cy5 pair with Cy5 in position-2, and 
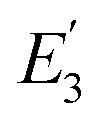
 is the FRET efficiency of the QD/Cy5 pair with Cy5 in position-3. The calculated results are as follows: 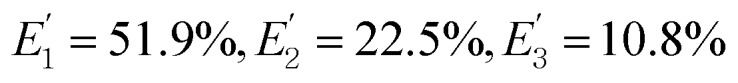, and the total FRET efficiency is theoretically estimated to be 85.1%, consistent with the value obtained experimentally by fluorescence spectroscopy measurement (84.9%) and the single QD-based nanosensor (85.3%), further confirming the formation of the QD-dsDNA-Cy5 nanosensor with multilayer of multiple Cy5 molecules.

**Fig. 3:**
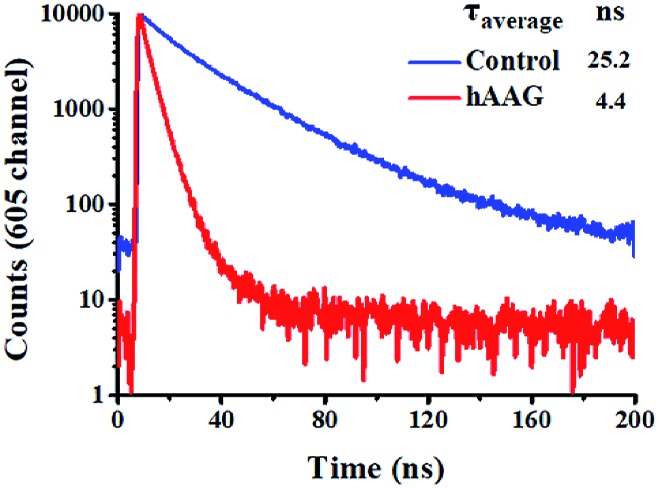
Fluorescence lifetime curves of the QD in the absence (control, blue line) and presence of hAAG (red line). The lifetime was measured in the emission channel of 605 nm. The QD concentration is 5 nM.

To verify efficient FRET between the QD and Cy5 in the QD-dsDNA-Cy5 nanostructure, we further measured the fluorescence lifetime of the QD (Fig. 3). The average lifetime (*τ*) of the QD in the control group without hAAG is 25.2 ns, whereas the *τ* of the QD is reduced to 4.4 ns in the presence of 0.1 U μL^–1^ hAAG. The FRET efficiency is calculated to be 82.5% according to eqn (7)7
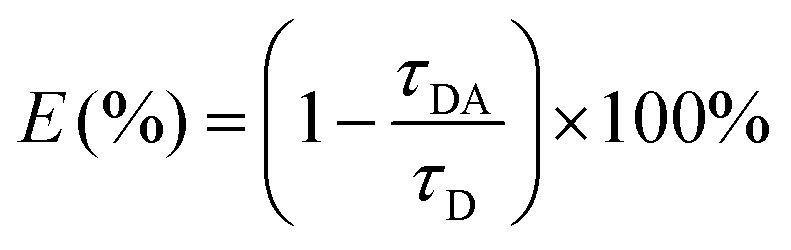
where *τ*_D_ is the fluorescence lifetime of the QD alone, and *τ*_DA_ is the fluorescence lifetime of the QD in the presence of hAAG. This value is close to that obtained by fluorescence spectroscopy measurement (84.9%) and the single QD-based nanosensor (85.3%).

### Measurement of hAAG activity by single-molecule imaging

**Fig. 4:**
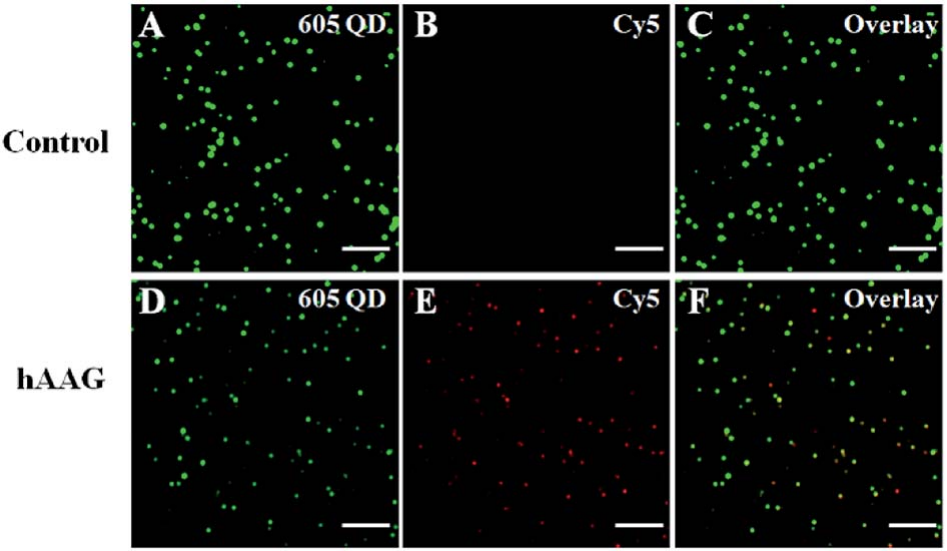
Single-molecule imaging of 605QD (A and D) and Cy5 (B and E) in the absence (A–C) and presence of hAAG (D–F). The 605QD fluorescence signals are shown in green (A and D), and the Cy5 fluorescence signals are shown in red (B and E). The colocalization of 605QD and Cy5 signals is indicated by yellow color (C and F). The hAAG concentration is 0.1 U μL^–1^, and the 605QD concentration is 25 pM. The scale bar is 5 μm.

We employed the single QD-based nanosensor to measure hAAG activity. In the control group without hAAG, only the 605QD signals are observed in the donor channel (Fig. 4A), without Cy5 fluorescence signals observed in the acceptor channel (Fig. 4B). When hAAG is present, both the 605QD fluorescence signals (Fig. 4D) and the Cy5 fluorescence signals (Fig. 4E) are observed simultaneously as a result of FRET from the 605QD to Cy5 in the QD-dsDNA-Cy5 nanostructure, with the yellow signals indicating the colocalization of 605QD and Cy5 (Fig. 4F). The near-zero background signal observed in the negative control (Fig. 4B) is crucial for the sensitive detection of hAAG activity. Notably, the fluorescence intensity of 605QD in the presence of hAAG (Fig. 4D) is much lower than that of 605QD in the absence of hAAG (Fig. 4A) due to efficient FRET from the 605QD to Cy5, but the number of QDs remains almost unchanged. Therefore, the simple quantification of Cy5 counts can be used for accurate measurement of hAAG activity. In addition, we used transmission electron microscopy (TEM) to characterize the obtained QD-dsDNA-Cy5 nanostructures (see ESI, Fig. S10†). The observed single QD with good dispersion clearly indicates the formation of the single QD-based nanosensor.

### Detection sensitivity

**Fig. 5:**
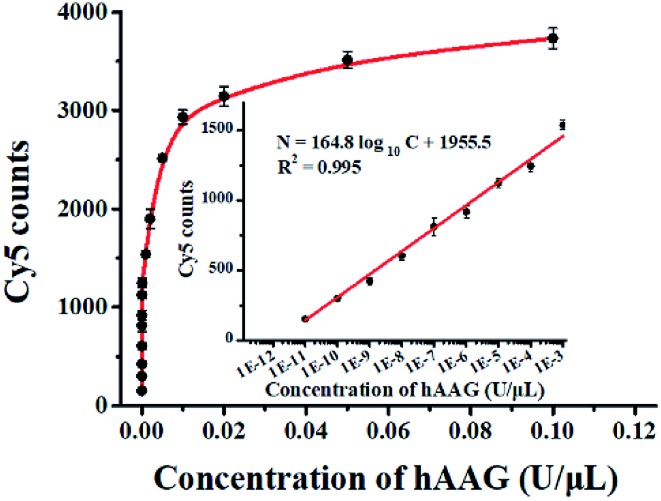
Measurement of Cy5 counts in response to variable concentrations of hAAG. The inset shows the linear relationship between Cy5 counts and the logarithm of hAAG concentration in the range from 1 × 10^–11^ to 1 × 10^–3^ U μL^–1^. Error bars represent standard deviations of three experiments.

To evaluate the sensitivity of the single QD-based nanosensor, we measured the Cy5 counts in response to variable concentrations of hAAG under the optimal experimental conditions (see ESI, Fig. S4–S7†). As shown in Fig. 5, when the concentration of hAAG increases from 1.0 × 10^–11^ to 0.1 U μL^–1^, the Cy5 counts enhance correspondingly. In the logarithmic scale, the Cy5 counts exhibit a linear correlation with the concentration of hAAG in the range from 1 × 10^–11^ to 1 × 10^–3^ U μL^–1^ (inset of Fig. 5). The regression equation is *N* = 164.8 log_10_ *C* + 1955.5 (*R*^2^ = 0.995), where *C* represents the concentration of hAAG (U μL^–1^) and *N* represents the Cy5 counts. The detection limit is calculated to be 4.42 × 10^–12^ U μL^–1^ based on the principle of the control group plus three times the standard deviation. The sensitivity of the single QD-based nanosensor has improved by as much as 7 orders of magnitude compared with that of the magnetic nanoparticle-based separation approach (1 × 10^–4^ U μL^–1^),^14^ and hyperbranched signal amplification-based fluorescent assay (9 × 10^–5^ U μL^–1^),^17^ and is 203-fold higher than that of ensemble fluorescence measurement (Fig. 2). The improved sensitivity can be attributed to (1) the specific hAAG-induced hypoxanthine excision repair,^43^,^44^ (2) the generation of large amounts of primers induced by APE1-mediated amplification, (3) the formation of the QD-dsDNA-Cy5 nanosensor with the multilayer of multiple Cy5 molecules, and (4) the near-zero background and high signal-to-noise ratio of single-molecule detection.^33^,^35^

### Detection selectivity

**Fig. 6:**
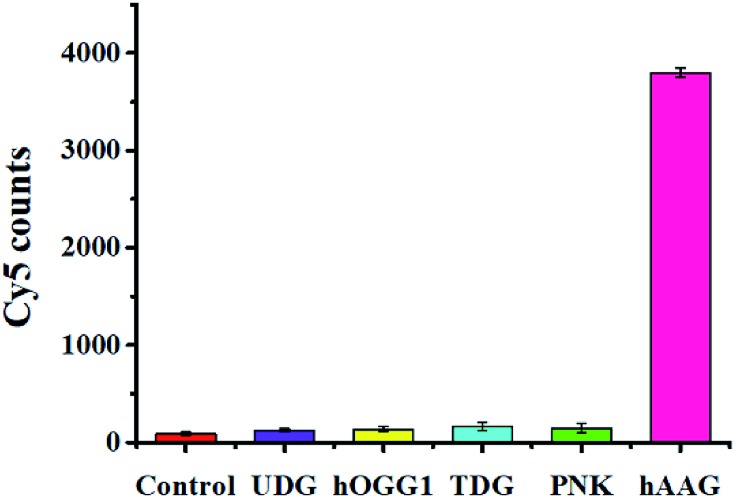
Measurement of Cy5 counts in response to the reaction buffer (control, red column), 0.2 U μL^–1^ UDG (violet column), 0.2 U μL^–1^ hOGG1 (yellow column), 0.2 U μL^–1^ TDG (cyan column), 0.2 U μL^–1^ PNK (green column), and 0.1 U μL^–1^ hAAG (pink column). Error bars represent standard deviations of three experiments.

To evaluate the selectivity of the single QD-based nanosensor, we used T4 polynucleotide kinase (PNK) and three DNA glycosylases including human 8-oxoguanine-DNA glycosylase 1 (hOGG1), uracil DNA glycosylase (UDG), and thymine DNA glycosylase (TDG) as the interference enzymes. PNK can catalyse the transfer of phosphate from the gamma position of adenosine triphosphate to the 5′-hydroxyl group of the DNA substrate.^40^ The hOGG1 can remove the damaged 8-hydroxyguanine (8-oxoG) from 8-oxoG/C base pairs in dsDNA and hydrolyze the 3′-phosphodiester bond of the abasic site. UDG can remove the uracil base from DNA and generate an abasic site by catalysing the hydrolysis of the N-glycosidic bond between deoxyribose and the uracil base. TDG can selectively remove T from G/T mismatches through the DNA BER pathway.^5^ In theory, none of these interference enzymes can recognize and remove hypoxanthine from the hairpin probe substrate. As shown in Fig. 6, a high Cy5 signal is observed in response to hAAG (pink column, Fig. 6), while no significant Cy5 signal is detected in the presence of reaction buffer (red column, Fig. 6), UDG (violet column, Fig. 6), hOGG1 (yellow column, Fig. 6), TDG (cyan column, Fig. 6), and PNK (green column, Fig. 6). This can be explained by the fact that only hAAG can generate the biotin-/multiple Cy5-labeled dsDNAs which can assemble on the surface of the 605QD to obtain the QD-dsDNA-Cy5 nanostructure with the multilayer of multiple Cy5 molecules. These results clearly demonstrate the excellent selectivity of the single QD-based nanosensor towards hAAG.

### Kinetic analysis

**Fig. 7:**
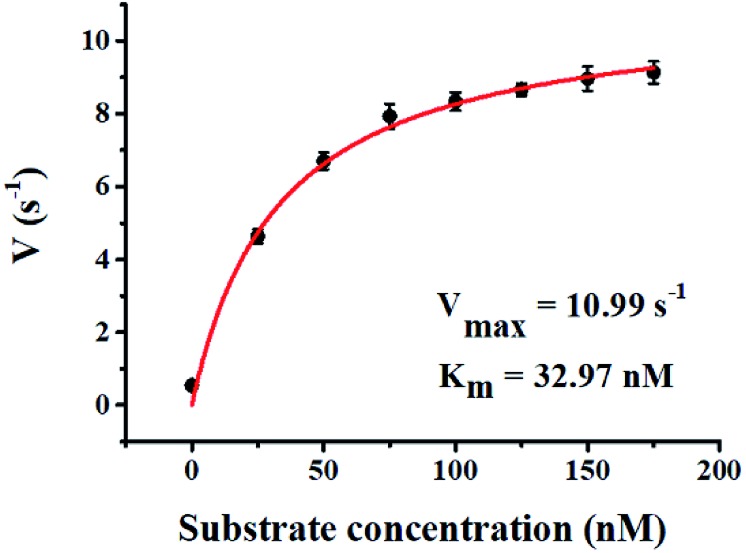
Variance of initial velocity (*V*) with various concentrations of the hairpin probe substrate. The hAAG concentration is 0.1 U μL^–1^. Error bars represent standard deviations of three experiments.

We used the single QD-based nanosensor to measure the kinetic parameters of hAAG by incubating 0.1 U μL^–1^ hAAG with 1 U of APE1 and varying concentrations of the hairpin probe substrate in 5 min reaction at 37 °C. The enzyme kinetic parameters of hAAG are obtained by fitting the experimental data to the Michaelis–Menten equation:8
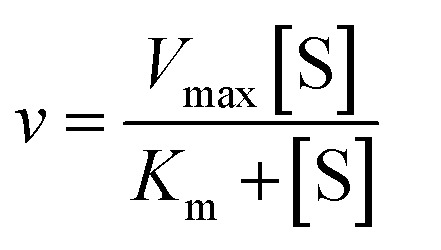
where *V*_max_ represents the maximum initial velocity, [S] represents the concentration of the hairpin probe substrate, and *K*_m_ is the Michaelis–Menten constant. As shown in Fig. 7, the initial velocity of hAAG enhances with increasing concentration of the hairpin probe substrate. The *V*_max_ is evaluated to be 10.99 s^–1^ and *K*_m_ is calculated to be 32.97 nM for hAAG. The *K*_m_ value is consistent with that obtained by the radioactive assay (13–42 nM).^47^ These results demonstrate that the proposed method can accurately determine the kinetic parameters of hAAG.

### Inhibition assay

**Fig. 8:**
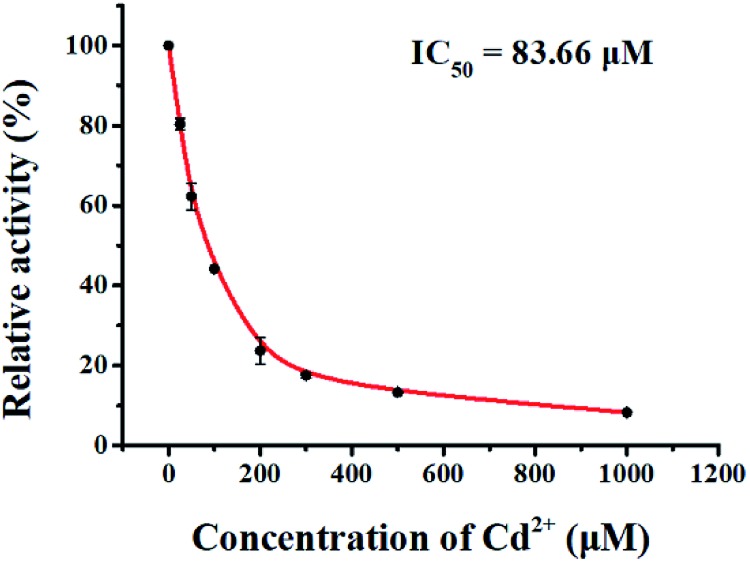
Measurement of the relative activity of hAAG in response to different concentrations of CdCl_2_. The hAAG concentration is 0.1 U μL^–1^. Error bars represent standard deviations of three experiments.

We used cadmium (CdCl_2_) as a model inhibitor of hAAG to demonstrate the feasibility of the single QD-based nanosensor for the inhibition assay. CdCl_2_ can inhibit the activity of hAAG towards a DNA oligonucleotide containing 1,*N*^6^-ethenoadenine (εA) and hypoxanthine (I), and it exhibits a metal-dependent inhibitory effect on hAAG catalytic activity at concentrations of 50–1000 μM.^48^,^49^ We measured the relative activity of hAAG in response to different concentrations of CdCl_2_, and we found that the relative activity of hAAG decreased with increasing concentration of CdCl_2_. The IC_50_ value is the inhibitor concentration required to reduce enzyme activity by 50%. The IC_50_ value is determined to be 83.66 μM (Fig. 8), which is smaller than the value of hAAG alone measured by the radioactive assay (120 μM).^48^ This can be explained by the fact that CdCl_2_ inhibits the nuclease activity of APE1 in the range of 10–100 μM (ref. 50) and the inhibition of APE1 by CdCl_2_ contributes to the inhibition of whole reaction.

### Detection of cellular hAAG activity

**Fig. 9:**
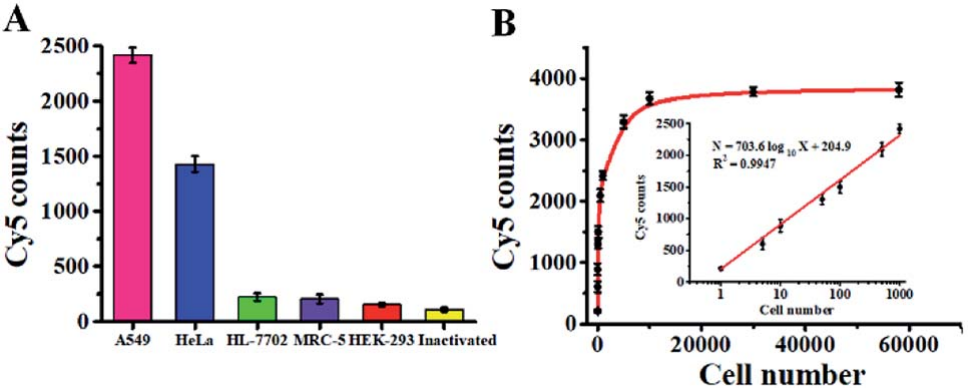
(A) Measurement of Cy5 counts in response to cell extracts (equivalent to 10^3^ cells) of A549 cells (pink column), HeLa cells (blue column), HL-7702 cells (green column), MRC-5 (violet column), HEK-293 (red column), and the inactivated A549 cell extracts (yellow column). (B) Measurement of Cy5 counts in response to different numbers of A549 cells. The inset shows the linear relationship between Cy5 counts and the logarithm of the number of A549 cells. Error bars represent standard deviations of three experiments.

Accurate detection of intracellular hAAG is essential for clinical diagnosis and treatment. To demonstrate the capability of the single QD-based nanosensor for real biological sample analysis, we measured hAAG activity in both cancer and normal cells. The cancer cells include the human lung adenocarcinoma cell line (A549 cells) and human cervical carcinoma cell line (HeLa cells), and the normal cells include the human hepatocyte cell line (HL-7702 cells), normal human lung cell line (MRC-5 cells) and human embryonic kidney cell line (HEK-293 cells). As shown in Fig. 9A, fewer Cy5 counts are measured in HL-7702 cells (Fig. 9A, green column), MRC-5 cells (Fig. 9A, violet column), and HEK-293 cells (Fig. 9A, red column), just little higher than those measured in the control group with the inactivated A549 cell extracts (Fig. 9A, yellow column), indicating the lack of hAAG in normal cells. In contrast, more Cy5 counts are measured in cancer cells including A549 cells (Fig. 9A, pink column) and HeLa cells (Fig. 9A, blue column), indicating the presence of hAAG in A549 cells and HeLa cells, consistent with previous research.^14^,^17^,^49^ These results demonstrate that this single QD-based nanosensor can be used to distinguish cancer cells from normal cells.

We further used A549 cells as a model to investigate the relationship between the Cy5 counts and cell number. As shown in Fig. 9B, the Cy5 counts enhance with increasing number of A549 cells in the range from 1 to 5.8 × 10^4^ cells. In the logarithmic scale, the Cy5 counts exhibit a linear correlation with the number of A549 cells from 1 to 1000 cells (inset of Fig. 9B), and the corresponding equation is *N* = 703.6 log_10_ *X* + 204.9 (*R*^2^ = 0.9947), where *N* is the Cy5 counts and *X* is the number of A549 cells. The detection limit is calculated to be 0.67 cell based on the evaluation of the average response of the negative control plus 3 times the standard deviation, indicating the feasibility of the single QD-based nanosensor for the detection of hAAG at the single-cell level.

## Conclusions

In summary, we developed a single QD-based nanosensor with multilayer of multiple acceptors for ultrasensitive detection of DNA repair enzyme hAAG. Through a three-step reaction including hAAG-specific cleavage of the hairpin probe, APE1-mediated cyclic cleavage and DNA polymerase-assisted amplification, multilayered Cy5 molecules can be assembled onto a single QD, significantly enhancing the FRET efficiency and improving the detection sensitivity. This single QD-based nanosensor has significant advantages as follows: (1) the introduction of endonuclease APE1 eliminates the design of a complex sequence with restriction sites for signal amplification; (2) the formation of the single QD-based nanosensor with the multilayer of multiple Cy5 molecules is confirmed theoretically and experimentally, significantly amplifying the FRET efficiency; (3) the introduction of single molecule detection with a high signal-to-noise ratio and near zero background, greatly improving the detection sensitivity. The sensitivity of the single QD-based nanosensor (detection limit of 4.42 × 10^–12^ U μL^–1^) has improved by 7 orders of magnitude compared with that of the magnetic nanoparticle-based separation approach (1× 10^–4^ U μL^–1^)^14^ and hyperbranched signal amplification-based fluorescent method (9 × 10^–5^ U μL^–1^).^17^ Notably, this method can even detect hAAG in 1 single cancer cell and distinguish the cancer cells from the normal cells. In addition, this single QD-based nanosensor can be used for the kinetic study and inhibition assay, holding great potential for further applications in biomedical research, drug discovery and clinical diagnosis. Importantly, this single QD-based nanosensor may combine with appropriate DNA substrates to become a universal platform for the detection of various DNA repair enzymes.

## Experimental

### Materials

All oligonucleotides (Table 1) purified by HPLC were obtained from Sangon Biotechnology Co. Ltd. (Shanghai, China). Human alkyladenine DNA glycosylase (hAAG), human apurinic/apyrimidinic endonuclease 1 (APE1), Klenow fragment (3′ → 5′ exo^–^), human 8-oxoguanine-DNA glycosylase 1 (hOGG1), uracil DNA glycosylase (UDG), T4 polynucleotide kinase (PNK), 10× NEBuffer 2 (500 mM NaCl, 100 mM Tris–HCl, 100 mM MgCl_2_, 10 mM DTT, pH 7.9), and 10× NEBuffer 4 (500 mM potassium acetate, 200 mM Tris–acetate, 100 mM magnesium acetate, 10 mM DTT, pH 7.9) were purchased from New England Biolabs (Ipswich, MA, USA). Thymine DNA glycosylase (TDG) was bought from R&D System (Minneapolis, MN, USA). RNase-free water, dCTP, dGTP and dTTP were purchased from TaKaRa Biotechnology Co. Ltd. (Dalian, China). Cyanine 5-dATP (Cy5-dATP) was obtained from PerkinElmer (Foster City, CA, USA). The streptavidin-coated CdSe/ZnS quantum dots with a maximum emission of 605 nm (605QD) were purchased from Invitrogen Corporation (Carlsbad, CA, USA). The human lung adenocarcinoma cell line (A549 cells), human cervical carcinoma cell line (HeLa cells), human hepatocyte cell line (HL-7702 cells), normal human lung cell line (MRC-5 cells), and human embryonic kidney cell line (HEK-293 cells) were purchased from the Cell Bank of Chinese Academy of Sciences (Shanghai, China). Other reagents were of analytical grade and used just as received without further purification.

**Table 1:** Sequences of the oligonucleotides^*a*^

Note	Sequence (5′–3′)
Hairpin probe substrate	CAC GAT GAA TCC TAG ACT ATT TTT ATA GTC TAG GAT TC**I[combining low line]** TCG TGA CAA TAC AAC –NH_2_
AP probe	CGC TGG AGC TGA GTT GTT GTA T**X[combining low line]**G TCA CGA –NH_2_
Capture probe	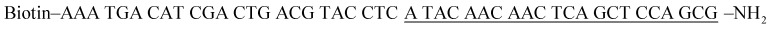
Cy5-labeled hairpin probe	CAC GAT GAA TCC TAG ACT ATT TTT ATA GTC TAG GAT TC**I[combining low line]** TCG TGA CAA TAC AAC-Cy5
Trigger	TCG TGA CAA TAC AAC –NH_2_
Primer	CGC TGG AGC TGA GTT GTT GTA T

^*a*^The underlined bold letter “I” is deoxyinosine, and the underlined bold letter “X” represents an AP site. In the capture probe, the underlined letters may hybridize with the primer.

### Preparation of the hairpin probe

The 10 μM hairpin probe was incubated in the buffer containing 150 μM MgCl_2_ and 1 mM Tris–HCl (pH 8.0) at 95 °C for 5 min, followed by slowly cooling to room temperature to form the hairpin structure. The obtained probes were stored at 4 °C for further use.

### Enzyme reaction and the formation of the QD-dsDNA-Cy5 nanostructure

Enzyme reaction involves three consecutive steps. First, the 0.48 μM annealed hairpin probe was incubated in 10 μL of reaction solution containing variable concentration of hAAG, 1 U of APE1 and 1× NEBuffer 4 at 37 °C for 1 h. Second, 1.44 μL of 10 μM AP probe and 2 U of APE1 were added to the solution, and the mixture was incubated at 37 °C for 40 min, followed by at 95 °C for 20 min. Third, the amplification reaction was carried out in 25 μL of solution containing 0.576 μM capture probe, 8 μM Cy5-dATP, 200 μM dGTP, 200 μM dCTP, 200 μM dTTP, 2 U of Klenow fragment, and 1× NEBuffer 2 at 37 °C for 90 min in the dark. The reaction was terminated by heating at 75 °C for 20 minutes. Then enzyme reaction products and the 605QDs with a final concentration of 5 nM were incubated in 80 μL of solution containing QD incubation buffer (3 mM MgCl_2_, 100 mM Tris–HCl, and 10 mM (NH_4_)_2_SO_4_, pH 8.0) at room temperature for 15 min to form the QD-dsDNA-Cy5 nanostructure.

### Gel electrophoresis and fluorescence measurement

The DNA products were analyzed using a Bio-Rad ChemiDoc MP Imaging System (Hercules, CA, USA). The products stained with SYBR Gold were analyzed by 12% polyacrylamide gel electrophoresis (PAGE) in TBE buffer (44.5 mM Tris-boric acid, 1 mM EDTA, pH 8.2) at a 110 V constant voltage for 50 min. The fluorescent DNA fragments of the enzyme reaction products were analyzed using an illumination source of Epi-green (460–490 nm excitation) and a 516–544 nm filter for SYBR Gold fluorophores, and an illumination source of Epi-red (625–650 nm excitation) and 675–725 nm filter for Cy5 fluorophores. The fluorescence signals of reaction products were measured using an F-7000 spectrometer (Hitachi, Japan) equipped with a xenon lamp as the excitation source. The excitation wavelength was 405 nm, and the spectra were recorded in the range from 550 to 750 nm. Both the excitation and emission slits were set to 5.0 nm. The fluorescence intensities at 605 nm (the maximum emission of QDs) and 670 nm (the maximum emission of Cy5) were used for data analysis. The fluorescence lifetime of QDs was measured using an FLS1000 (Edinburgh Instruments, UK).

### Measurement of melting curves

For the melting curve assay, the amplification products were analyzed using a Bio-Rad CFX Connect real-time system (Hercules, CA, USA) with SYBR Green I as the indicator, and the fluorescence intensity was monitored at intervals of 30 s. The 50 nM capture probe, 50 nM primer, 10 μM dATP, 250 μM dGTP, 250 μM dCTP, 250 μM dTTP and 2 U of Klenow fragment were incubated at 37 °C for 1 h to obtain the amplification products. The pre-amplification products only include the 50 nM capture probe and 50 nM primer. Each sample was monitored at temperatures of 50 °C–95 °C. The specific melting temperature was defined as the inflexion point at which –d*F*/d*T* reaches a maximum (where *F* is the fluorescence intensity and *T* is the temperature).

### Single-molecule detection and data analysis

In single-molecule measurement, the reaction products were diluted 200-fold in QD incubation buffer. The 10 μL of samples were put on a coverslip for TIRF microscopy (Nikon, Ti-E, Japan) imaging. The 405 nm laser was used to excite the 605QDs. The photons from the 605QD and Cy5 were collected by an oil immersion 100× objective (Nikon, Japan), and were split up into the 605QD channel (573–613 nm filter) and Cy5 channel (661.5–690.5 nm filter) by a dichroic mirror, and were imaged by a digital CMSO EMCCD camera (Hamamatsu Photonics K. K., Japan) with an exposure time of 500 ms. For data analysis, a region of interest of 600 × 600 pixels was selected for Cy5 molecule counting by using Image J software. The number of Cy5 was obtained by calculating ten frames.

### Inhibition assay

For the hAAG inhibition assay, variable concentrations of CdCl_2_ were added to the glycosylase reaction mixture, followed by the above three-step reaction. The relative activity of hAAG (*RA*) was measured according to eqn (9):9
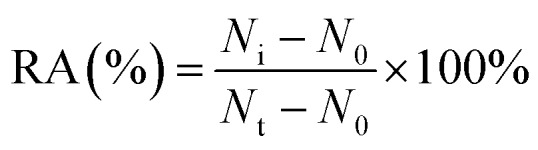
where *N*_0_ is the Cy5 counting number when hAAG is absent, *N*_t_ is the Cy5 counting number when hAAG is present, and *N*_i_ is the Cy5 counting number in the presence of both hAAG and CdCl_2_. The IC_50_ value was calculated from the curve of RA *versus* the CdCl_2_ concentration.

### Cell culture and preparation of cell extracts

Different cell lines including A549 cells, HeLa cells, HL-7702 cells, MRC-5 cells and HEK-293 cells were cultured in Dulbecco's modified Eagle medium (DMEM; Invitrogen, USA) containing 10% fetal bovine serum (FBS; Gibco, USA) and 1% penicillin–streptomycin (Invitrogen, USA). The cells were cultured in a humidified incubator with 5% CO_2_ at 37 °C. The nuclear extracts were collected using the nuclear extract kit (ActiveMotif, Carlsbad, CA, USA) according to the manufacturer's protocol. The obtained supernatant was subjected to the hAAG activity assay.

## Conflicts of interest

There are no conflicts to declare.

## Supplementary Material

Supplementary information
