# Supplementary material for: A single quantum dot-based nanosensor with multilayer of multiple acceptors for ultrasensitive detection of human alkyladenine DNA glycosylase
Source: Chem Sci. 2019 Aug 6;10(37):8675–84. doi: 10.1039/c9sc02137j (PMC6849492; doi:10.1039/c9sc02137j)
Supplement: Supplementary file 1 [file SC-010-C9SC02137J-s001.pdf]

Electronic Supplementary Material (ESI) for Chemical Science.
This journal is © The Royal Society of Chemistry 2019

Electronic Supplementary Information

Single quantum dot-based nanosensor with multilayer of multiple acceptors for ultrasensitive detection of human alkyladenine DNA glycosylase

Chen-chen Li,[‡] Wan-xin Liu,[‡] Juan Hu* and Chun-yang Zhang*

College of Chemistry, Chemical Engineering and Materials Science, Collaborative Innovation Center of Functionalized Probes for Chemical Imaging in Universities of Shandong, Key Laboratory of Molecular and Nano Probes, Ministry of Education, Shandong Provincial Key Laboratory of Clean Production of Fine Chemicals, Shandong Normal University, Jinan 250014, China.

* Corresponding author. Tel.: +86 0531-86186033; Fax: +86 0531-82615258. E-mail: cyzhang@sdnu.edu.cn; juanhu@sdnu.edu.cn.

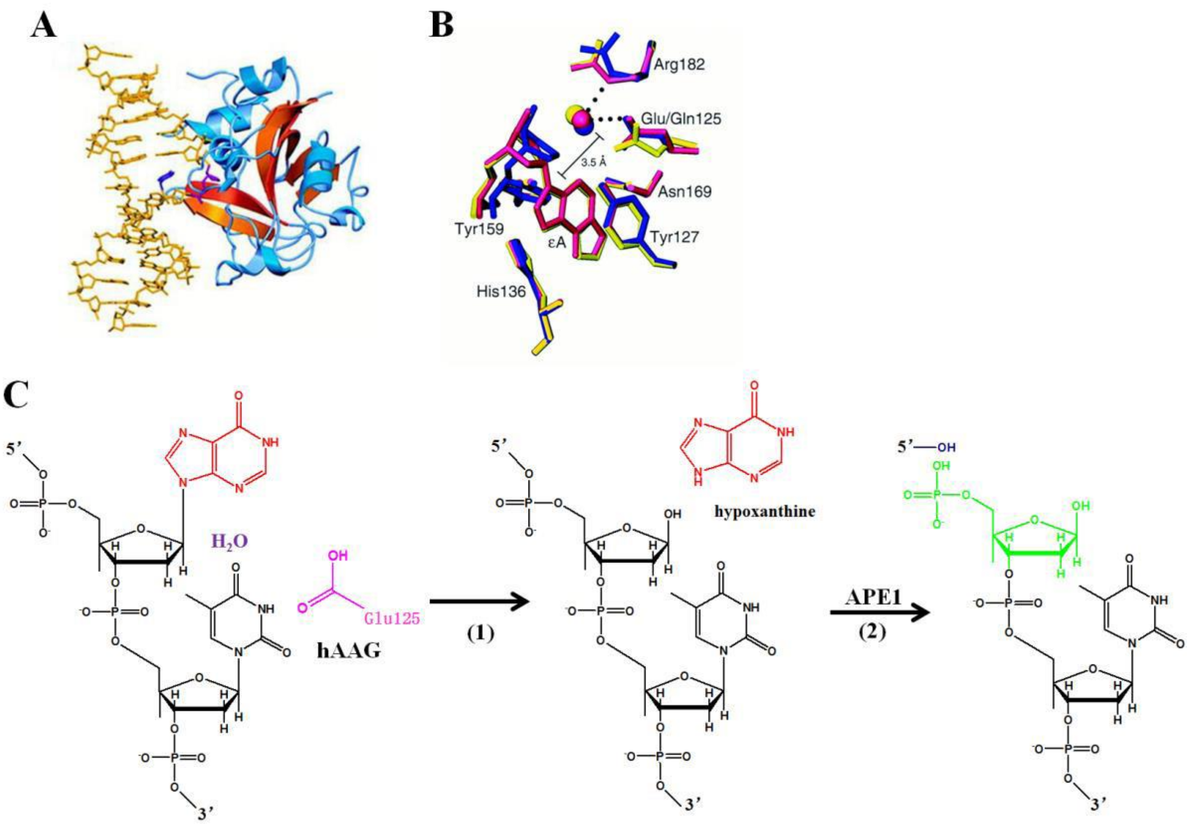

Figure S1. (A) Structure of hAAG bounding to DNA substrate.[1] (B) Structure of the active site of hAAG.[2] (C) Molecular mechanism of the hAAG-actuated hypoxanthine-excision repair reaction of hairpin probe substrate in the presence of APE1. The reaction involves two steps: (1) the removal of hypoxanthine base and the formation of AP site, and (2) the cleavage of the phosphodiester bond on the 5' side at the AP site.

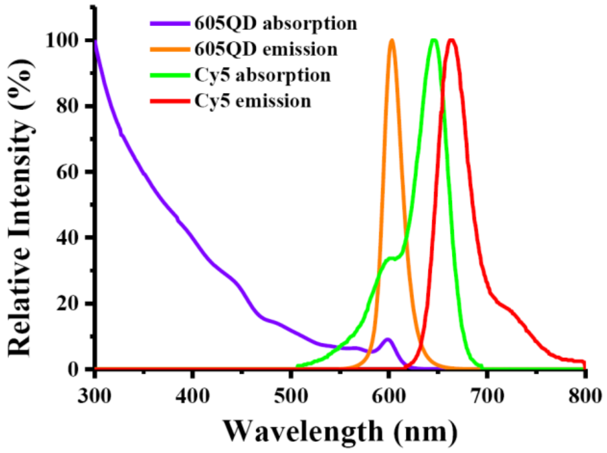

Figure S2. The absorption and emission spectra of 605QD and Cy5. Purple line, absorption spectrum of 605QD; Orange line, emission spectrum of 605QD; Green line, absorption spectrum of Cy5; Red line, emission spectrum of Cy5. The 605QD/Cy5 as a FRET pair permits only minimal spectral cross-talk. The broad absorption spectrum of

the QD allows its excitation at 405 nm, which is near zero absorption of Cy5, efficiently eliminating direct acceptor excitation. Thus, the wavelength of 405 nm is chosen as the excitation light.

Study the absorption of Cy5-dATP on the QD surface

We used a biotinylated random sequence (5'-GCC AGT GTA GTT GGA GCT GTG GCG TAG GCA AGA GTG TC-biotin-3') to investigate whether there is nonspecific absorption of Cy5-dATP (free Cy5) on the QD surface. The biotinylated random sequence is used to replace the capture probe in Scheme 1. As shown in Figure S3, the QD exhibits a distinct emission peak at the wavelength of 605 nm (Figure S3, black line). The Cy5 molecules cannot be excited by the excitation wavelength of 405 nm, and a near zero fluorescence signal is observed (Figure S3, green line). No Cy5 signal is detected in the control group without hAAG (Figure S3, blue line). In the presence of the biotinylated random sequence and hAAG, neither the hybridization of the released primers with the biotinylated random sequence nor the DNA polymerase-assisted amplification reaction occurs, and no Cy5 molecule is bound to the QD. Consequently, neither significant decrease in fluorescence intensity at 605 nm (QD signal) nor significant increase in fluorescence intensity at 670 nm (Cy5 signal) is observed (Figure S3, red line), suggesting no FRET from the QD to Cy5 and no nonspecific absorption of Cy5-dATP (free Cy5) on the surface of QD in the presence of the biotinylated random sequence and hAAG. Our results indicate that the Cy5 molecules can covalently bind to the QD surface only through the DNA polymerase-assisted amplification reaction and specific biotin-streptavidin binding in the presence of hAAG (Figure 1D) and there is no nonspecific absorption of Cy5-dATP on the surface of streptavidin-coated QDs (Figure S3) due to the presence of biotin-labeled probes (e.g., the capture probe in Scheme 1 and the biotinylated random sequence).

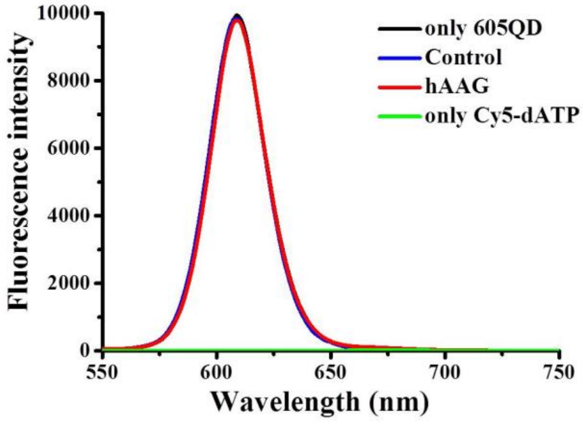

Figure S3. Measurement of 605QD and Cy5 fluorescence emission spectra in presence of the biotinylated random sequence (5'-GCC AGT GTA GTT GGA GCT GTG GCG TAG GCA AGA GTG TC-biotin-3') without hAAG (control, blue line) and with hAAG (red line). The 0.1 U/μL hAAG, 5 nM 605QD and 2.5 μM Cy5-dATP were used in this experiment. The excitation wavelength is 405 nm.

Optimization of the ratio of capture probe to QD.

To achieve the best performance of FRET, we investigated the influence of the capture probe-to-QD ratio upon FRET efficiency. Various concentrations of capture probe and primer were employed to form the biotin-/multiple Cy5-labeled dsDNA. The resultant dsDNAs were incubated at room temperature for 15 min with a fixed amount of QDs, and the fluorescence signals of both QDs and Cy5 were measured by single-molecular imaging. The FRET efficiency (*E*) in the single-QD-based nanosensor is obtained based on equation 1:

$$E\ (\%) = \left(1 - \frac{F_{DA}}{F_D}\right) \times 100\% = (1 - \frac{\sum I_{DA}}{\sum I_D}) \times 100\% \qquad (1)$$

where $\sum I_{DA}$ is the sum of 605QD intensity in the presence of Cy5 acceptor, and $\sum I_D$ is the sum of 605QD intensity in the absence of Cy5 acceptor. Both the FRET efficiency (Figure S4, blue line) and the Cy5 counts (Figure S4, red line) enhance with the increasing capture probes-to-QD ratio from 6:1 to 36:1, followed by the leveling off of both FRET efficiency and Cy5 counts beyond the ratio of 36:1, indicating that the biotin-binding sites on the surface of the QD have been saturated. The obtained capture probes-to-QD ratio of 36:1 is within the theoretically calculated biotin-binding sites per QD of 36-45. Because each QD is conjugated with 12-15 streptavidins, and each streptavidin conjugated to QD has three available biotin-binding sites. In theory, there are 36 to 45 biotin-binding sites per QD. Therefore, the ratio of capture probes-to-QD of 36:1 is used in the subsequent experiments.

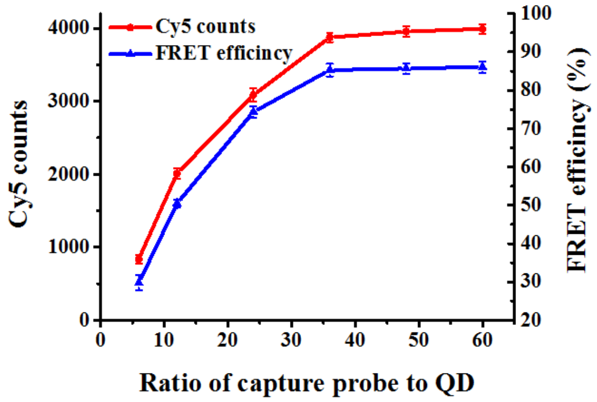

Figure S4. Variance of Cy5 counts (red curve) and FRET efficiency (blue curve) with the capture probes-to-QD ratio. The concentration of 605QD is 25 pM. Error bars represent standard deviations of three experiments.

Optimization of the hAAG-actuated hypoxanthine excision repair reaction

In the step of the hAAG-actuated hypoxanthine excision repair reaction, hairpin probe substrates are consumed. Therefore, the concentration of hairpin probe should be optimized. With a fixed concentration of AP probe (960 nM), we monitored the variance of Cy5 counts in response to different concentrations of hairpin probe (Figure S5). As shown in Figure S5, the Cy5 counts enhance with the increasing concentration of hairpin probe and reaches a plateau at the concentration of 480 nM. Thus, 480 nM hairpin probe is used in the subsequent research.

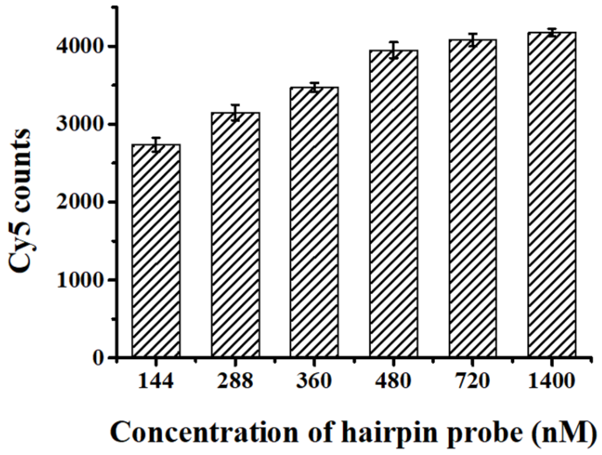

Figure S5. Variance of Cy5 counts with the different concentrations of hairpin probe. The hAAG concentration is 0.1 U/μL, and the concentration of AP probe is 960 nM. Error bars represent standard deviations of three experiments.

Optimization of the APE1-mediated circular-cleaving SDA

APE1 is used to cut the AP site in AP probe/trigger dsDNA in the APE1-mediated circular-cleaving SDA, leading to the breakage of AP probe and the generation of primer. We optimized the amount of APE1 in step 2. Figure S6 shows that the Cy5 counts enhance with the increasing amount of APE1, and reach the maximum value at the amount of 2 U. Therefore, 2 U of APE1 is used in the subsequent research.

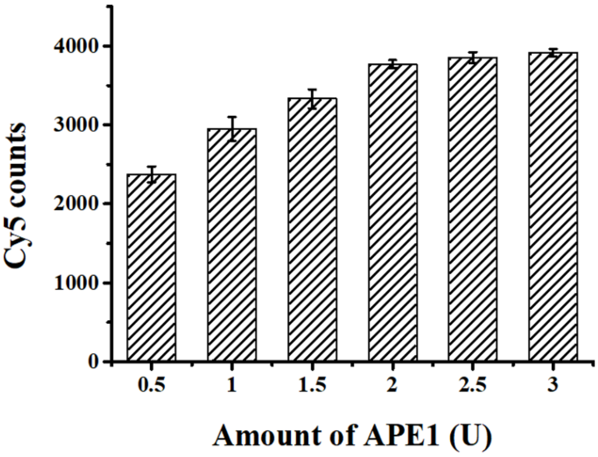

Figure S6. Variance of Cy5 counts with different amounts of APE1 in the APE1-mediated circular-cleaving SDA. The hAAG concentration is 0.1 U/μL. Error bars represent standard deviations of three experiments.

Optimization of the DNA polymerase-assisted amplification

The concentration of Cy5-dATP, the amount of Klenow Fragment and incubation time are essential to the DNA polymerase-assisted amplification reaction. To optimize the concentration of Cy5-dATP, we measured the Cy5 counts in response to various concentrations of Cy5-dATP. As shown in Figure S7A, the Cy5 counts enhance with the increase of Cy5-dATP concentration from 2 to 8 μM, and reach a plateau at the concentration of 8 μM. The more amount of Cy5-dATP may result in higher primer extension efficiency and the assembly of more Cy5 molecule onto the surface of QD and consequently the generation of more Cy5 counts. However, when the Cy5-dATP concentration is saturated for the primer extension reaction, there is no significant improvement in Cy5 counts. Thus, 8 μM Cy5-dATP is used in the subsequent research.

We further optimized the amount of Klenow Fragment of DNA polymerase-assisted amplification reaction. As shown in Figure S7B, the Cy5 counts enhance with the increasing amount of Klenow Fragment from 1 to 2 U, and level off beyond 2 U. Thus, 2 U of Klenow Fragment is used in the subsequent research.

To determine the optimized incubation time of DNA polymerase-assisted amplification reaction, we measured the Cy5 counts with the incubation time from 30 to 130 min (Figure S7C). The Cy5 counts enhance with the reaction time from 30 to 90 min and reach a plateau beyond 90 min. Thus, amplification reaction time of 90 min is used in the subsequent research.

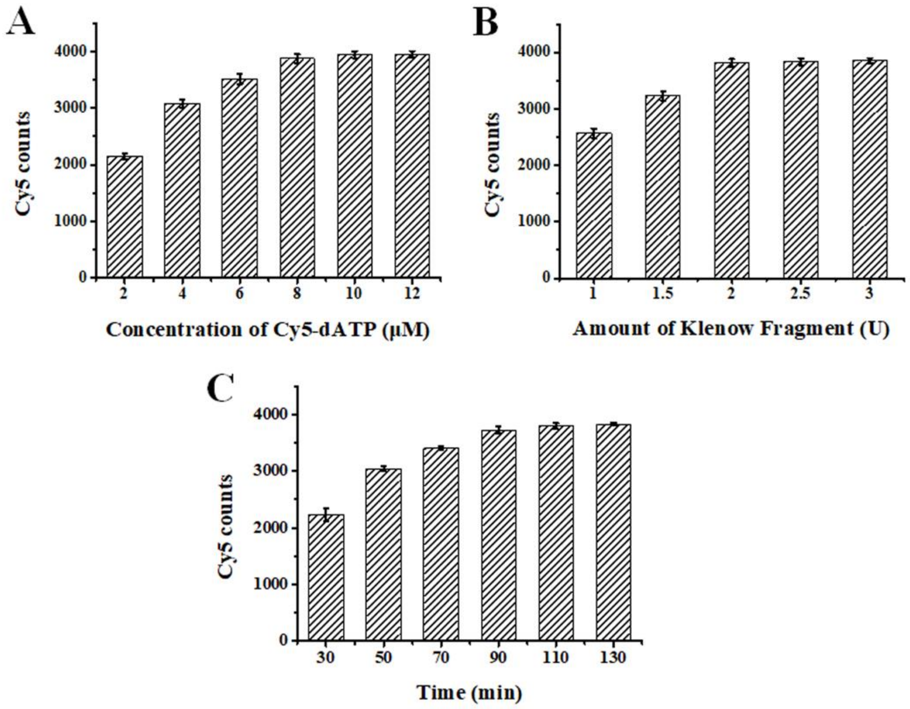

Figure S7. (A) Variance of Cy5 counts with different concentrations of Cy5-dATP. (B) Variance of Cy5 counts with different amounts of Klenow Fragment. (C) Variance of Cy5 counts with the amplification time. The hAAG concentration is 0.1 U/μL. Error bars represent standard deviations of three experiments.

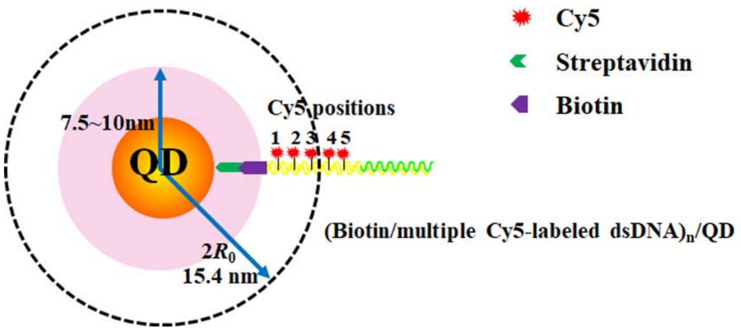

Figure S8. Schematic illustration of an idealized streptavidin-functionalized 605QD donor with multilayered Cy5 acceptors. The FRET efficiency of the QD/Cy5 pair with Cy5 in position-1-3 constitutes the overall efficiency

because only Cy5 molecules in position-1-3 are within an efficient distance of $2R_0$.

Calculation of the number of biotin-/multiple Cy5-labeled dsDNAs per 605QD

As shown in Figure S9, the number of biotin-/multiple Cy5-labeled dsDNAs per 605QD is calculated using fluorescence emission spectra. First, we calculated the number of Cy5 molecules per biotin-/multiple Cy5-labeled dsDNA. After the steps 1-3 (Scheme 1) were performed in the presence of capture probes (14.4 pmol), the obtained biotin-/multiple Cy5-labeled dsDNAs were incubated with the streptavidin-coated magnetic beads (0.2 mg, Invitrogen Corporation). The free Cy5-dATPs were removed by a magnetic field. Then the magnetic bead-coupled biotin-/multiple Cy5-labeled dsDNAs were incubated with 20 µL of NEBuffer 4 (50 mM potassium acetate, 20 mM Tris-acetate, 10 mM magnesium acetate, 1 mM DTT, pH 7.9) containing 20 U of exonuclease III at 37 °C for 1 h to release the Cy5 molecules. The supernatant solution containing the released Cy5 molecules was subjected to fluorescence spectra measurement at the excitation wavelength of 630 nm (Figure S9A). The number of Cy5 molecules in the biotin-/multiple Cy5-labeled dsDNAs (i.e., the released Cy5 molecules from the magnetic beads) is estimated to be 72.43 pmol according to the calibration curve in Figure S9D. The average number of Cy5 molecules per biotin-/multiple Cy5-labeled dsDNA is calculated to be 72.43 pmol / 14.4 pmol = 5.

We further calculated the number of biotin-/multiple Cy5-labeled dsDNAs per 605QD. The quantity of 605QD was kept constant (0.4 pmol), and different ratio of capture probe to 605QD was used in the experiments. After the conjugation of biotin-/multiple Cy5-labeled dsDNAs with the 605QD, the free biotin-/multiple Cy5-labeled dsDNAs in the solution were captured by the streptavidin-coated magnetic beads. At the capture probe-to-605QD ratio of 48, the number of the released Cy5 molecules from the magnetic beads (Figure S9B) is estimated to be 24.77 pmol according to the calibration curve in Figure S9D. Taking into account five Cy5 molecules per dsDNA, the quantity of the biotin-/multiple Cy5-labeled dsDNAs assembled on the 605QDs is calculated to be 19.2 pmol – 24.77 pmol / 5 = 14.25 pmol, and the average number of biotin-/multiple Cy5-labeled dsDNA per 605QD is calculated to be 14.25 pmol / 0.4 pmol = 36 (Figure S9C). The calculated average number of biotin-/multiple Cy5-labeled dsDNA per 605QD is in good agreement with that obtained by the FRET experiment (Figure S4).

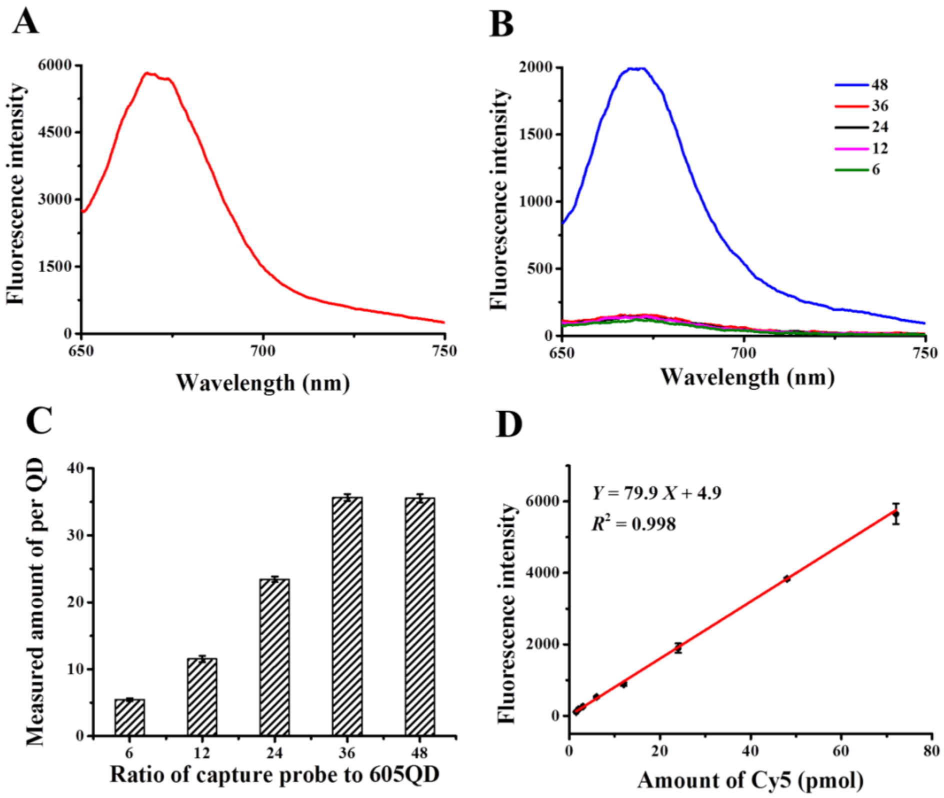

Figure S9. (A) Fluorescence spectra of the released Cy5 molecules from the streptavidin-coated magnetic beads. (B) Fluorescence spectra of the released Cy5 molecules from the streptavidin-coated magnetic beads as a function of the capture probes-to-QD ratio. (C) The measured amount of biotin-/multiple Cy5-labeled dsDNA per 605QD as a function of the capture probes-to-QD ratio. (D) Variance of fluorescence intensity as a function of the amount of Cy5 molecules (Cy5-dATP).

Transmission electron microscope (TEM) images of single QD-based nanosensor

We employed TEM to observe the streptavidin-functionalized QDs. As shown in Figure S10A, the streptavidin-functionalized QDs were well dispersed. We further used TEM to characterize the obtained QD-dsDNA-Cy5 nanostructures (Figure S10B). The observed single QD with well dispersion clearly indicates the formation of single QD-based nanosensor.

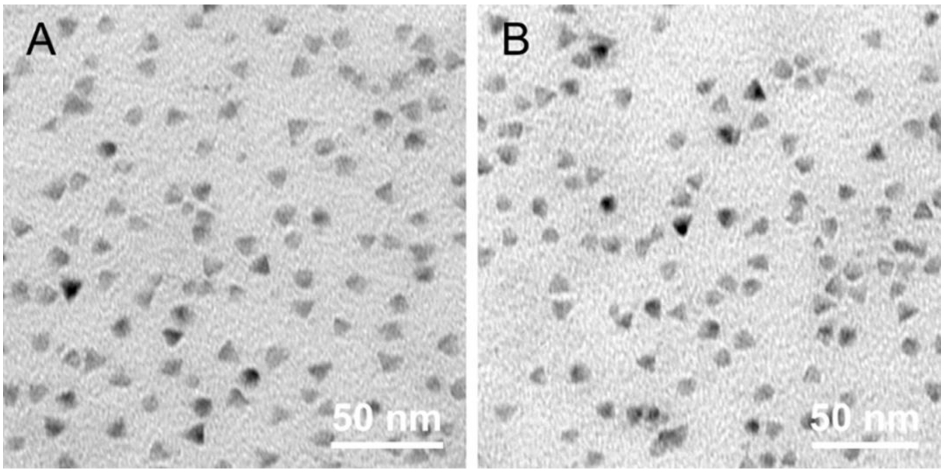

Figure S10. TEM images of streptavidin-functionalized QDs (A) and the obtained QD-dsDNA-Cy5 nanostructures (B). The TEM images were obtained by Hitachi TEM system (Hitachi, Japan).

References

1 A. Y. Lau, O. D. Scharer, L. Samson, G. L. Verdine and T. Ellenberger, *Cell*, 1998, **95**, 249-258.

2 A. Y. Lau, M. D. Wyatt, B. J. Glassner, L. D. Samson and T. Ellenberger, *Proc. Natl. Acad. Sci. U. S. A.*, 2000, **97**, 13573-13578.
